# Patient-centered education in dyslipidemia management: a systematic review

**DOI:** 10.2478/abm-2022-0026

**Published:** 2023-06-16

**Authors:** Farhana Fakhira Ismail, Adyani Md Redzuan, Chong Wei Wen

**Affiliations:** Centre for Quality Management of Medicine, Faculty of Pharmacy, Universiti Kebangsaan Malaysia, Kuala Lumpur 50300, Malaysia; Department of Pharmacy Practice, Faculty of Pharmacy, Universiti Teknologi MARA, Puncak Alam, Selangor 42300, Malaysia

**Keywords:** cardiovascular diseases, disease management, patient education as topic, patient participation, pharmacists, practice patterns

## Abstract

**Background:**

Dyslipidemia management is crucial to reduce mortality and morbidity from cardiovascular diseases (CVDs). Patients must be educated and empowered to enable them to manage their own diseases. Various methods of patient education, such as patient-centered education (PCE) or non-PCE (such as didactic education or any traditional form of education), have been implemented.

**Objective:**

To review and determine the effectiveness of PCE for dyslipidemia management compared with usual care. The primary outcome chosen was cholesterol level. Other measures, such as psychosocial or cognitive, behavioral, and other relevant outcomes, were also extracted. Additionally, underlying theories and other contributing factors that may have led to the success of the intervention were also reviewed and discussed.

**Methods:**

We conducted searches in PubMed, Cumulative Index to Nursing and Allied Health Literature (CINAHL), Scopus, and Google Scholar from inception until April 2021. All studies involving randomized controlled trials were included. Study quality was assessed using the Critical Appraisal Skills Program (CASP) checklist specifically for randomized controlled trials.

**Results:**

The search identified 8,847 records. Of these, 20 studies were eligible for inclusion. Interventions using a PCE approach were largely successful. Contributing factors extracted from the included studies were underlying theories, instant reward system, dietary education, collaborative care, duration of intervention with systematic follow-ups, social support, adherence assessment method, and usage of e-health.

**Conclusions:**

PCE is successful in achieving the desired outcomes in dyslipidemia management. Future studies may incorporate the elements of PCE to improve the management of dyslipidemia in hospital or community settings where appropriate.

Cardiovascular disease (CVD) is associated with morbidity and mortality worldwide [1]. A study conducted in 2019 across 13 countries [2] found that 1 in 3 adults had been diagnosed with CVD. Based on WHO data in 2019, an estimated 17.9 million people died from CVDs, representing 32% of all global deaths. There is strong evidence linking dyslipidemia with CVD [3]. Dyslipidemia is a known primary risk factor for CVD in children and adolescents [4]. The prevention and appropriate management of dyslipidemia would significantly impact cardiovascular morbidity and mortality [5]. Due to its asymptomatic nature, the management of dyslipidemia must be strategized and addressed by health-care professionals. Synchronized contributions from all parties, including patients and health-care professionals, have been proposed to improve patients’ clinical outcomes and subsequently reduce the burden of CVD [6].

Many articles have highlighted the importance of self-management among patients with chronic conditions such as CVD. For example, in their study, Grady and Gough [7] asserted that self-management is crucial in treating chronic conditions. The authors also mentioned that self-management emphasized the patient’s responsibility to manage their disease while guided by their health-care professionals. This stance is supported by findings from Bosworth et al. [8], which highlighted that the self-management strategy is effective and should be integrated as a crucial component in providing high-quality care to patients with CVD [8].

For patients with chronic disease to be successful in self-management, they must first be informed and empowered [9]. This approach will allow patients to adopt preventive and curative steps in managing their conditions [10]. The relationship between self-management and patient empowerment has been explored and discussed in many articles. Rappaport [11] defines empowerment as a process by which people gain mastery over their affairs. The empowerment process involves the provision of knowledge, skills, and responsibility to patients, which will lead to behavioral change and potentially improve their overall health [10].

In their review, Bosworth et al. [8] describe the concept of self-management in managing patients with hypertension and heart diseases. The authors have proposed 5 significant factors to ensure the success of self-management, including patient-centered programs [8]. Exploring the concept of patient-centeredness, Stewart et al. [12] proposed 6 dimensions of patient-centered care, which are as follows: (1) exploring both the disease and illness experience; (2) understanding the whole person; (3) finding common ground; (4) incorporating prevention and health promotion; (5) enhancing the patient–doctor relationship; and (6) being realistic. All these dimensions may be incorporated in patient-centered education (PCE). Comparing PCE with didactic education, the latter mainly involves a patient as a passive recipient receiving standardized information from health-care professionals [9]. In this situation, the patient is expected to passively adhere to the instructions and advice given by the health-care professional [9]. By contrast, in PCE, patients are treated with deep respect, listened to, and actively involved in their own plans of treatment, with their wishes being honored throughout their disease management journey [13].

Different types of education may affect patients differently and act as a critical component in determining the success of self-management. Effective patient education will then lead to behavioral change and may improve the patient’s health generally. To our knowledge, there is no published review to study the effectiveness of PCE in dyslipidemia management. Therefore, this systematic review aims to analyze the effectiveness of PCE in dyslipidemia management in comparison with usual care. In addition, we also examine the underlying theories and other elements of patient-centered care, which may contribute to the success of the interventions.

## Methods

### Literature search

We conducted a systematic review of the published literature in accordance with the Preferred Reporting Items for Systematic Review and Meta-Analyses (PRISMA) guidelines [14]. We searched the literature in 3 databases, namely PubMed, Cumulative Index to Nursing and Allied Health Literature (CINAHL), and Scopus, from inception to April 2021. Additional articles were sought by reviewing references of eligible studies and conducting a search on Google Scholar. Studies were identified with the following search terms: (“patient-centered education” OR synonyms) AND (“dyslipidemia” OR synonyms). We included both medical subject heading (MeSH) terms and free-text terms. Full search terms are shown in **Table 1**. A filter was applied to the searches on the databases to restrict retrieval of off-target articles, whereby non-English articles were excluded.

**Table 1.** Search strategy for the databases

**Number**	**Keyword†**
1	Dyslipidemia
2	Hyperlipidemia
3	Hypercholesterolemia
4	Hypertriglyceridemia
5	1 OR 2 OR 3 OR 4
6	Patient centered (text word)
7	Patient centered education
8	Patient education
9	Patient empowerment
10	6 OR 7 OR 8 OR 9 OR 10
11	6 AND 10

† Here, OR and AND are Boolean algebraic operators.

### Study selection

By adopting the population, interventions, comparisons, and outcomes (PICO) framework as a template, the inclusion and exclusion criteria presented in **Table 2** were applied to set limits to the scope of the review. The population (P) aspect focused on patients with dyslipidemia, which commonly presents with CVD. The intervention (I) was PCE. In determining whether the interventions were patient-centered or not, the definition of patient-centered care by Robinson et al. [15] was adopted. They described the fundamental characteristics of patient-centered care, which are patient involvement in the care and individualization of patient care.

**Table 2.** Inclusion and exclusion criteria for articles

**Inclusion criteria**	**Exclusion criteria**
✓ English articles only	➢ Review article (systematic review and meta-analysis)
✓ RCT	➢ Articles with study protocol only
✓ Published from inception till April 2021	➢ Articles with poor randomization method
✓ Included patient education as the intervention	➢ Articles with unclear description of patient education
✓ Intervention used PCE with the following criteria:	
– Applied SDM (the decision is mutually agreed by the patients and health-care professionals)	
– Mentioned patient-centered approaches such as MI	
✓ Articles assessed by the CASP checklist for assessment of study quality	

CASP, Critical Appraisal Skills Programme for RCTs [16]; MI, motivational interviewing; PCE, patient-centered education; RCT, randomized controlled trial; SDM, shared decision-making.

In terms of patient involvement, studies were only included when the authors clearly described active participation from the patients. Some studies described that the patients were actively involved in the care plan, while some studies also mentioned specifically the usage of shared decision-making (SDM). SDM is a method when the patients and health-care professionals make decisions together [17]. The SDM approach aims to ensure that decisions are made based on the patient’s. values and preferences.

Regarding individualization, the education provided in the study must be targeted according to patients’ specific needs. Garvey et al. [18] supported this fundamental characteristic of patient-centered care when describing that one of the goals of patient-centered care is the development of an individualized treatment plan. A study was also included in the review when the researchers used other established patient-centered approaches such as motivational interviewing (MI). Elwyn et al. [17] asserted that MI and SDM are patient-centered methods, and both have been associated with significant improvement in patient outcomes.

These interventions were compared against the usual care (C), such as a didactic or traditional form of education. Finally, the outcome (O) measure in this review is the cholesterol level or any other psychosocial, cognitive, or behavioral outcome reported in the studies. Articles retrieved from the database search were then exported to a reference managing software EndNote X9 (Clarivate), and duplicates were omitted. Because our focus was on randomized controlled trials (RCTs), only studies with the keyword “trial” were included using the smart screening tool in EndNote. The eligible studies underwent preliminary screening by 2 authors independently. The authors screened both titles and abstracts for potential relevance in line with the PICO criteria. Articles that met the predefined eligibility criteria were included for full-text screening and were assessed independently by the 2 authors. Any disagreements between the authors regarding study inclusion were resolved through discussion and consensus with the third author. The inclusion and exclusion criteria are listed in **Table 2**.

### Data extraction

The key characteristics and pertinent information from the eligible studies were extracted and documented. The extracted information included author, year of publication, study location, study design, sample size, patient demographic, and study setting, which are summarized in **Table 3**. Other information collected were methods of intervention such as the health-care professionals involved, intervention duration, elements involved in the intervention group, and the underlying theories or models being used, summarized in **Table 4**. The key mechanisms were retrieved if no theories or models were presented by the studies. The outcomes of the intervention were also extracted and are summarized in **Table 5**. These included psychosocial, cognitive, or behavioral outcomes, cholesterol level, and cardiometabolic outcomes, such as blood pressure (BP), weight, body mass index (BMI), and glycated hemoglobin A (HbA1c). Low-density lipoprotein (LDL) reduction was also compared between the intervention and control groups and is reported in **Table 5**. For studies that did not measure LDL, the difference in total cholesterol (TC) or triglyceride (TG) level was extracted and reported. Significant information, such as *P*-value, odds ratio (OR), relative risk (RR), and confidence interval (CI), was also included whenever reported. All values were reported in the intervention group in comparison with the usual care. Other reported outcomes that did not fit any of the criteria mentioned earlier, but were relevant in dyslipidemia management, were also extracted and are described in **Table 5**.

**Table 3.** Summary of studies included

**Study’s first author, year**	**Study location**	**Design**	**Sample size**	**Patient demographic**	**Setting**
Eaton (2011) [3]	New England	2-arm RCT	4,105	Patients with regular follow-up and interested in coronary heart disease risk reduction	Primary care clinic
Goyer (2012) [6]	Canada	3-arm RCT	185	Patients with at least 2 cardiovascular risk factors	Montreal Clinical Research Institute
McDermott (2012) [19]	The United States	3-arm RCT	355	Peripheral arterial disease patients with LDL-C ≥ 70 mg·dL^−1^	Medical institution (university)
Ockene (1999) [20]	The United States	3-arm RCT	1,162	Patients with blood TC level in the highest 25th percentile and had a previous scheduled visit	Community health center (health maintenance organization)
Fortin (2016) [21]	Canada (North America)	3-arm RCT	664	Patients aged 18–75 years with at least 1 chronic conditions or risk factors	Primary care practice
Lear (2002) [22]	Canada (North America)	2-arm RCT	302	Patients with ischemic heart disease	Hospital
Lin (2012) [23]	The United States	2-arm RCT	214	Patients with poorly controlled diabetes and coronary heart diseases	Hospital (medical center)
Allen (2011) [24]	The United States	2-arm RCT	525	Patients with established CVD and LDL-C/BP/HbA1c exceeding target goal	Community health center
Babazono, (2007) [25]	Japan	2-arm RCT	99	Patients with high SBP/DBP/HbA1c during annual health checkup	Health center
Bosworth (2018) [26]	The United States	2-arm RCT	428	Patients with poorly controlled hypertension and/or hypercholesterolemia	Medical center/hospital
Byrne (2020) [27]	The United Kingdom	2-arm RCT	212	Patients prescribed with statins and had TC ≥5 mmol·L^−1^	Primary care center
Ho (2014) [28]	The United States	2-arm RCT	241	Patients admitted with acute coronary syndrome as the primary reason	Medical center (hospital)
Iturralde (2019) [29]	The United States	2-arm RCT	647	Patients with ≥1 uncontrolled CVD risk factors for at least 2 years before	Kaiser Permanente (non-profit integrated healthcare delivery system)
Jarab (2012) [30]	Jordan	2-arm RCT	156	Follow-up patients with type 2 diabetes	Hospital
Jiang (2007) [31]	China	2-arm RCT	167	Patients who were first hospitalized with either angina pectoris or myocardial infarction	Hospital
Maindal (2014) [32]	Denmark	2-arm RCT	509	Patients aged 40–69 years at the time of screening and diagnosis of screening-detected type 2 diabetes	Primary care clinic
Mok (2013) [33]	Hong Kong	2-arm RCT	82	Patients diagnosed with myocardial infarction	Hospital
Sol (2008) [34]	The Netherlands	2-arm RCT	154	Referred patients with symptomatic vascular diseases	Hospital
Zhang (2019) [35]	China	2-arm RCT	62	Patients with history of cardiometabolic syndrome	Medical university-affiliated hospital
Daumit (2020) [36]	The United States	2-arm RCT	269	Patients with at least 1 cardiovascular risk factor	Community outpatient clinic

BP, blood pressure; CVD, cardiovascular diseases; DBP, diastolic blood pressure; HbA1c, glycated hemoglobin A; LDL-C, low density lipoprotein-cholesterol; RCT, randomized controlled trial; SBP, systolic blood pressure; TC, total cholesterol.

**Table 4.** Summary of interventions

**First author, year**	**Interventions by**	**Intervention duration**	**Interventions**	**Patient-centered elements**	**Control**	**Theories/models/key mechanism**
Eaton (2011) [3]	Physician	12 months	4 academic detailing sessions Physicians received patient education toolkit, a computer kiosk with patient activation software, and PDA-based decision support tool Patients were guided via interactive SDM aided by the PDA decision support tool	Active patient involvement in care plan Individualized care plan	Physicians received PDA only without the decision support tool and no patient education toolkit	Theory: chronic care model
Goyer (2012) [6]	Nutritionist Psychologist Kinesiologist Nurse Physician	3 months	12 weekly group sessions of 3 h between Months 3 and 6 of the study Follow-up sessions every 3 months until the end of the second 2-year protocol	Active patient involvement in care plan Individualized care plan MI	Management was left to the family physician. Patients were called after 1 year for address verification and reminder for the 2-year follow up Called for the 2-year assessment	Theory/model: health belief model, Prochaska stages of change
McDermott (2012) [19]	Health counselor	12 months	Patient-centered counseling for medication adherence and recommendation to visit the physicians Telephone calls every 6 weeks for 12-month duration advising about medication adherence and encouragement to increase walking activity	Active patient involvement in care plan Individualized care plan	Second control arm: - 8 telephone calls delivered every 6 weeks - No attempts for behavior change Third control arm: - No scheduled telephone calls	Key mechanism: health-care professional–patient relationship to promote patient activation (patient requested more-intensive lipid-lowering therapy from their physicians)
Ockene (1999) [20]	Physician	12 months	Physicians received nutrition counseling training with office support program Physicians delivered patient-centered and interactive nutrition counseling assisted with office support program Office support program helped the physicians to provide counseling by providing all necessary materials	Active patient involvement in care plan Individualized care plan	Second control arm: - Physicians received nutrition counseling training only Third control arm: - Usual care (not being described further)	Theory: social learning theory
Fortin (2016) [21]	Nurse CDPM professional	3 months	Self-management support, patients’ education about risk assessment, and lifestyle changes assisted with printed materials Collaborative care The intervention group received the intervention right away after the baseline measurement	Active patient involvement in care plan Individualized care plan MI	Second control arm: - Received similar intervention as intervention group but 3 months after baseline (delayed intervention) Third control arm: - Received no intervention at all for 1 year	Key mechanism: health-care professional–patient relationship to promote self-management, empowerment, and self-efficacy
Lear (2002) [22]	Dietitian Exercise specialist nurse	48 months	6 CRPs, 6 telephone follow-ups, 3 lifestyle and risk factor counseling sessions annually and continued for 2 years Patients were counseled about behavior changes and guided to develop individualized goal setting	Active patient involvement in care plan Individualized care plan	Return to their family physician’s care and come to the study clinic only to undergo annual outcome assessment Copy of the laboratory results were sent to the participants’ family physicians	Theory: transtheoretical theory, social cognitive theory
Lin (2012) [23]	Nurse Physician	12 months	Patient education by nurses, followed by regular follow-up Weekly caseload reviews by physician consultants Monitoring was done by visits or telephone calls initially 2–3 times a month	Active patient involvement in care plan Individualized care plan	Patients were advised to consult their primary care physicians Patients can self-refer or be referred for specialty services, including mental health	Theory: chronic care model
Allen (2011) [24]	Nurse Community health worker	12 months	Patient education followed by follow-ups. Follow-up frequency depends on participants’ progress Progress reviewed by community health worker Each follow-up session discussed individualized patients’ goals, barriers, strategies, and support to aid patients in achieving the goal	Active patient involvement in care plan Individualized care plan MI	Received results of baseline with the recommended goal level Received a pamphlet on controlling risk factors from American Heart Association	Theory: chronic care model
Babazono (2007) [25]	Dietitian Health exercise instructor Public health nurse	12 months	Patient education about lifestyle changes Follow-up support, twice a year at patient’s home Health center visits for blood tests at the end of 4 and 6 months	Active patient involvement in care plan Individualized care plan	Received result of their blood tests and leaflets	Model: transtheoretical model
Bosworth (2018) [26]	Clinical pharmacist specialist	12 months	12 monthly telephone calls emphasizing on medication management, training on home BP monitor, encouragement of self-monitoring of blood glucose, adverse effect monitoring, and medication adherence	Active patient involvement in care plan Individualized care plan	Received primary care and CVD management according to the decision of the provider At baseline and 6 months, patients received generic printed educational material on ways for CVD risk reduction	Model: transtheoretical model
Byrne (2020) [27]	Facilitator (health-care professional)	12 months	2 education sessions with follow-up support involving 44 weeks of text messages and 2 telephone calls Session 1 focused on risk assessment and role of statin, meanwhile Session 2 focused on lifestyle modification and behavioral control techniques The text messages were automated and contained medication reminders as well as information and advice	Active patient involvement in care plan Individualized care plan	Received basic information leaflet Continued treatment with their usual general practitioner for primary prevention of CVD	Theory: behavior change wheel
Ho (2014) [28]	Pharmacist Primary care clinician/cardiologist	12 months	Patient education at 1 week and 1 month visit Collaborative care between pharmacist and patient’s primary care clinician and/or cardiologist 2 types of voice messaging (educational and medication refill reminder calls)	Patient involvement in care plan Individualized care plan	Scheduled for clinic visit after 1 year for risk assessment	Model: Wagner chronic care model, medication adherence model
Iturralde (2019) [29]	Nurse Pharmacist	12 months	Received usual care with group-based behavioral intervention 3 group-based patient activation sessions. These sessions included contacts with the nurses/pharmacists by secure message, telephone calls, or video appointments Development of individualized care plan Live demonstration of electronic patient portals and participants’ role play	Active patient involvement in care plan Individualized care plan MI	Received usual care Telephone follow-ups or secure messages through the electronic patient portal	Theory: chronic care model
Jarab (2012) [30]	Clinical pharmacist	6 months	Structured patient education and discussion, with provision of booklets Followed by 8 weekly telephone calls by clinical pharmacists. During telephone call, prescription was reviewed and the adherence to the treatment plan was discussed	Active patient involvement in care plan Individualized care plan MI	Received usual care by medical and nursing staff, included patient assessment at 3 and 6 months	Theory/key mechanism: health-care professional–patient relationship to promote patient’s self-management behavior
Jiang (2007) [31]	Nurse	3 months	12-week CRP divided into 2 phases, which were the hospital-based/family education and home-based rehabilitation care Involvement of family members in the hospital-based and home-based phases Follow-ups through home visits and telephone calls	Active patient involvement in care plan Individualized care plan	Received risk assessments together with intervention group at baseline, 3 months, and 6 months	Key mechanism: health-care professional–patient relationship in providing education to promote change in health behavior and physiological risk parameter
Maindal (2014) [32]	Nurse Dietitian Physiotherapist General physician	3 months	Received intensive treatment for behavioral change and pharmacological treatments from general practitioners. Also received invitation to take part in the intervention group 12-week patient-centered health education program with 2 individual counseling interviews 8 group sessions focused on action competence, CVD risk, and dietary advice according to individual goal	Active patient involvement in care plan Individualized care plan	Received intensive treatment for behavioral change and pharmacological treatments from general practitioners	Theory: motivation theory
Mok (2013) [33]	Nurse	2 months	8 weeks of nurse follow-up dietary intervention, including: face-to-face consultations, take-home self-management workbook, and fortnightly telephone follow-ups	Active patient involvement in care plan Individualized care plan	Outpatient medical follow-up by cardiologist Standard cardiac rehabilitation provided by hospital-Dietary class within 1 week after diagnosis of myocardial ischemia	Key mechanism: health-care professional–patient relationship to promote dietary change
Sol (2008) [34]	Nurse	12 months	Nursing care consisted of (1) self-efficacy promotion and (2) medical treatment of vascular risk factors. Patients were given information and tailored advice based on their conditions Patients were guided for individualized goal setting for lifestyle changes. Patients underwent regular follow-up for weight, BP, and fasting lipid and glucose levels	Active patient involvement in care plan Individualized care plan	Scheduled follow-up visit after 1 year for risk factor measurement	Key mechanism: health-care professional–patient relationship to promote self-efficacy and improvement in vascular risk factors
Zhang (2019) [35]	Psychologist Internal medicine specialist	3 months	24 workshops that applied SDM; partnership establishment; and patients were supported to have individualized goals	Active patient involvement in care plan (SDM) Individualized care plan	General information about cardiometabolic syndrome risk factors Sent weekly text messages. No in-person contacts other than the scheduled measurements	Key mechanism: Skinner behavior intensified techniques
Daumit (2020) [36]	Nurse Physician Health coach	18 months	Weekly individualized counseling sessions for the first 6 months and at least every 2 weeks thereafter Collaborative care among health coaches, nurses, and physicians Had point system to reward attendance and behavior change	Active patient involvement in care plan Individualized care plan MI	Had assessment during scheduled follow-up at 6 months and 18 months	Theory/model: behavioral self-management concepts, social cognitive theory, solution-focused therapy

BP, blood pressure; CDPM, chronic disease prevention and management; CRP, cardiac rehabilitation program; CVD, cardiovascular diseases; MI, motivational interviewing; PDA, personal digital assistant; SDM, shared-decision making.

**Table 5.** Summary of impact of interventions

**First author**	**Psychosocial/cognitive**	**Behavioral (smoking/physical activity/diet or medication adherence)**	**Cholesterol level**	**Other cardiometabolic outcomes (BP/weight/BMI/HbA1c)**	**Other outcomes**	**Difference in LDL level (reduction) in intervention and control groups**
Eaton [3]	–	–	0 LDL (95% CI, OR = 1.27) 0 non-HDL (95% CI, OR = 1.23)	–	–	–
Goyer [6]	+ Mental health status (*P*< 0.001)	+ Kilocalories intake (*P*= 0.022) + Physical activity (*P*< 0.001) 0 Smoking status	+ TC (*P*< 0.001) + TG (*P*= 0.047) 0 HDL + LDL (*P*= 0.046)	+ SBP (*P*< 0.001) + Weight (*P*< 0.001) + BMI (*P*< 0.001) + HbA1c 0 Waist circumference	+ Reduction in CVD risk score (Framingham Risk Score) (*P*< 0.005)	Difference in intervention group: 9.0 mg·dL^−1^ Difference in control group: 5.4 mg·dL^−1^
McDermott [19]	+ Patient activation (95% CI, *P*= 0.016) + Self-efficacy (95% CI, *P*< 0.001)	–	+ LDL (95% CI, *P*= 0.035)	–	+ Pharmacotherapy initiation and adjustments (95% CI, *P*< 0.001)	Difference in intervention group: 18.4 mg·dL^−1^ Difference in control group (usual care): 11.1 mg·dL^−1^
Ockene [20]	–	+ Reduction in consumption of saturated fats (*P*= 0.01)	0 TC (*P*= 0.07) 0 LDL (*P*= 0.10) 0 HDL (*P*= 0.09) 0 TG (*P*= 0.03)	+ Weight (*P*< 0.001) + BMI (*P*< 0.001)	–	Difference in intervention group: 1.98 mg·dL^−1^ Difference in control group (usual care): 0.18 mg·dL^−1^
Fortin [21]	–	+ Self-monitoring (95% CI, *P*= 0.001, RR = 2.40) + Emotional well-being (95% CI, *P*= 0.012, RR = 1.73) + Skill and technique acquisition (95% CI, *P*= 0.001, RR = 1.70) 0 Physical activity (95% CI, *P*= 0.276, OR = 3.81) 0 Fruit and vegetable consumption (95% CI, *P*= 0.198, OR = 2.36)	–	+ BMI (95% CI, *P*< 0.001)	–	–
Lear [22]	0 Self-efficacy 0 Perceived stress	0 Smoking status 0 Physical activity	0 TC 0 LDL 0 HDL 0 TG	+ BMI (*P*< 0.05) + Waist circumference (*P*< 0.05) 0 BP	+ Higher PTCA procedures (*P*< 0.05) + Less CABG procedures (*P*< 0.05)	–
Lin [23]	–	+ Glucose monitoring (*P*= 0.06, RR = 1.28) + BP monitoring (*P*< 0.001, RR = 3.20) 0 Medication adherence	–	–	+ Pharmacotherapy initiation and adjustment rates for antidepressants (*P*< 0.001, RR = 6.20) + Pharmacotherapy initiation and adjustment rates for insulin (*P*< 0.001, RR = 2.97) + Pharmacotherapy initiation and adjustment rates for antihypertensive medications (*P*< 0.001, RR = 1.86)	–
Allen [24]	+ Perceptions of the quality of chronic illness care (95% CI, *P*< 0.001)	–	+ TC (95% CI, *P*< 0.001) + LDL (95% CI, *P*< 0.001) + TG (95% CI, *P*= 0.013) 0 HDL (95% CI, *P*= 0.497)	+ SBP (95% CI, *P*= 0.003) + DBP (95% CI, *P*= 0.013) + HbA1c (95% CI, *P*= 0.034)	–	Difference in intervention group: 21.6 mg·dL^−1^ Difference in control group (usual care): 5.7 mg·dL^−1^
Babazono [25]	–	+ Number of steps per day (*P*< 0.001) + Vegetable intake (95% CI, *P*< 0.05, OR = 3.80) 0 Total calorie intake	0 TC 0 LDL 0 TG 0 HDL	0 BMI 0 BP 0 HbA1c	–	Difference in intervention group: 1.4 mg·dL^−1^ Difference in control group (usual care): increment of 0.1 mg·dL^−1^
Bosworth [26]	–	–	+ TC (95% CI, *P*= 0.03) 0 LDL 0 HDL (95% CI, *P*= 0.08)	0 SBP (*P*= 0.34) 0 DBP 0 HbA1c (95% CI) (*P*= 0.72)	–	Difference in intervention group: 9.7 mg·dL^−1^ Difference in control group (usual care): 8.9 mg·dL^−1^
Byrne [27]	+ Perceived control and understanding of the condition (95% CI, *P*< 0.027)	0 Medication adherence to statin (95% CI, *P*= 0.968, OR = 1.02) + Walking activity (95% CI, *P*< 0.001)	0 TC (95% CI, *P*= 0.120) 0 HDL (95% CI, *P*= 0.814)	0 SBP (95% CI, *P*= 0.096) + DBP (95% CI, *P*= 0.002) + Waist circumference (95% CI, *P*= 0.012) 0 BMI (95% CI, *P*= 0.088)	0 CVD risk score (95% CI, *P*= 0.165)	TC: difference in intervention group: 12.42 mg·dL^−1^ Difference in control group (usual care): 6.12 mg·dL^−1^
Ho [28]	–	+ Medication adherence (95% CI, *P*= 0.03)	0 LDL (*P*= 0.90)	0 SBP (*P*= 0.50) 0 DBP (*P*= 0.50)	–	Difference in intervention group: 13 mg·dL^−1^ Difference in control group (usual care): 12 mg·dL^−1^
Iturralde [29]	+ Patient activation (*P*= 0.01) + Patient-centered care (*P*= 0.003)	0 Statin adherence (*P*= 0.93)	0 LDL (*P*= 0.97)	0 SBP (*P*= 0.80) 0 HbA1c (*P*= 0.28)	0 1 year CVD risk factor + Engagement with the healthcare system using online tools (*P*= 0.01)	–
Jarab [30]	–	+ Medication adherence (self-report) (*P*= 0.003) + Self-care activities (*P*= 0.007)	+ LDL (*P*= 0.031, 95% CI) + TG (*P*= 0.017, 95% CI) 0 HDL (*P*= 0.728, 95% CI)	+ SBP (*P*= 0.035, 95% CI) + DBP (*P*= 0.026, 95% CI) + HbA1c (*P*= 0.019, 95% CI) 0 BMI (*P*= 0.189, 95% CI)	–	Difference in intervention group: 10.8 mg·dL^−1^ Difference in control group (usual care): 7.2 mg·dL^−1^
Jiang [31]	–	+ Medication adherence (at 3 months) (*P*= 0.029) 0 Medication adherence (at 6 months) (*P*= 0.143) + Walking activity (at 6 months) (*P*= 0.002) + Step 2 diet adherence (at 6 months) (*P*= 0.002) 0 Smoking status	+ TC (at 6 months) (*P*= 0.001) + TG (at 6 months) (*P*= 0.011) + LDL (at 6 months) (*P*= 0.001) 0 HDL (at 6 months) (*P*= 0.293)	+ SBP (at 3 months) (*P*= 0.021) 0 SBP (at 6 months) (*P*= 0.216) + DBP (at 3 months) (*P*= 0.030) 0 DBP (at 6 months) (*P*= 0.148) 0 Body weight (at 3 months) (*P*= 0.157) 0 Body weight (at 6 months) (*P*= 0.099)	–	Difference in intervention group: 8.1 mg·dL^−1^ Difference in control group (usual care): 2.7 mg·dL^−1^
Maindal [32]	+ Patient activation (*P*= 0.002, 95% CI)	0 Physical activity (*P*= 0.600, 95% CI) 0 Smoking status (*P*= 0.056, 95% CI)	+ TC (*P*= 0.027, 95% CI)	0 SBP (*P*= 0.372, 95% CI) 0 DBP (*P*= 0.140, 95% CI) 0 HbA1c (*P*= 0.371, 95% CI) 0 BMI (*P*= 0.831, 95% CI)	0- to 10-year CVD risk score (*P*= 0.878, 95% CI)	TC: difference between intervention and control groups: 4.32 mg·dL^−1^
Mok [33]	–	+ Reduction in consumption of saturated fats and salted food (*P*< 0.001) + Increased intake of heart-healthy foods (*P*< 0.001)	0 TC 0 TG + HDL (*P*= 0.001)	–	–	Difference in intervention group: no difference Difference in control group (usual care): increase 4.63 mg·dL^−1^
Sol [34]	0 Total self-efficacy + Self-efficacy in choosing healthy food (*P*= 0.01) + Self-efficacy in doing extra exercises (*P*= 0.03)	–	0 LDL (95% CI, OR = 0.95)	0 SBP (95% CI, OR = 1.07) 0 BMI (95% CI, OR = 0.93)	–	–
Zhang [35]	+ Quality of life (*P*< 0.001)	0 Physical activity 0 Smoking status	+TG (*P*< 0.001)	+ SBP (*P*< 0.001) + Waist circumference (*P*< 0.001)	–	TG: difference in intervention group: 14.4 mg·dL^−1^ Difference in control group (usual care): increase of 3.6 mg·dL^−1^
Daumit [36]	–	+ Smoking status (*P*= 0.004, 95% CI)	0 TC 0 HDL 0 LDL	0 SBP	+ Reduction in 10-year Framingham risk score (*P*= 0.02, 95% CI)	Difference in intervention group: 8.2 mg·dL^−1^ Difference in control group (usual care): 3.7 mg·dL^−1^

+ = Significant improvement (*P*< 0.05) compared with the control group.

0 = No significant effect of the intervention (*P*≥ 0.05) compared with the control group.

− = No result reported.

95% CI, 95% confidence interval reported in the article; BMI, body mass index; BP, blood pressure; CABG, coronary artery bypass graft; CI, confidence interval; CVD, cardiovascular disease; DBP, diastolic blood pressure; HbA1c, glycated hemoglobin A; HDL, high-density lipoprotein; LDL, low-density lipoprotein; OR, odds ratio; PTCA, percutaneous transluminal coronary angioplasty; RR, relative risk; SBP, systolic blood pressure; TC, total cholesterol; TG, triglyceride.

### Study quality assessment

The Critical Appraisal Skills Programme (CASP) [16]) for RCTs was adopted to evaluate the quality of the studies included. The CASP checklist consists of reviewing the RCT in terms of its basic study design (clarity of research question, appropriateness of randomization method), the methodology (blinding method, study groups), the results (effects of intervention being reported comprehensively, precision, benefits that the study brings), and in terms of the impact of the results to our targeted population. A score of 1 was assigned for each criterion if “yes” was the response, whereas a score of 0 was assigned if the response was “no” or “uncertain.” The review of quality assessment was conducted independently by 1 reviewer and was further assessed by a second reviewer to avoid the risk of bias. Any disagreements were resolved by consensus before finalizing the articles to be included in the study. The criteria assessments for all the included studies are summarized in **Table 6**.

**Table 6.** Criteria assessments for studies included

**Study’s first author**	**Does the study describe PCE?**	**1. Clearly focused research question**	**2. Was the assignment of participants randomized?**	**3. Were all participants accounted for at its conclusion?**	**4. Were the participants/investigators blinded to intervention?**	**5. Were the study groups similar at the start of RCT? -was a baseline set?-were any differences found between study groups that may affect outcome**	**6. Apart from the intervention, did each study group receive same level of care?**	**7. Were the effects of intervention reported comprehensively? - were power calculation, etc., reported**	**8. Was the precision of the estimate of the intervention or treatment effect reported? Were CIs reported?**	**9. Do the benefits of the intervention outweigh the harms and costs?**	**10. Can the results be applied to any local population?**	**11. Would the intervention provide greater value than any of the existing interventions?**
Eaton [3]	1	1	1	1	0	1	1	1	1	1	1	1
Goyer [6]	1	1	1	1	1	1	1	1	1	1	1	1
McDermott [19]	1	1	1	1	0	1	1	1	1	1	1	1
Ockene [20]	1	1	1	1	0	1	1	1	1	1	1	1
Fortin [21]	1	1	1	1	1	1	1	1	1	1	1	1
Lear [22]	1	1	1	1	1	1	1	1	1	1	1	1
Lin [23]	1	1	1	1	0	1	1	1	1	1	1	1
Allen [24]	1	1	1	1	0	1	1	1	1	1	1	1
Babazono [25]	1	1	1	1	0	1	1	1	1	1	1	1
Bosworth [26]	1	1	1	1	0	1	1	1	1	1	1	1
Byrne [27]	1	1	1	1	1	1	1	1	1	1	1	1
Ho [28]	1	1	1	1	1	1	1	1	1	1	1	1
Iturralde [29]	1	1	1	1	1	1	1	1	1	1	1	1
Jarab [30]	1	1	1	1	1	1	1	1	1	1	1	1
Jiang [31]	1	1	1	1	1	1	1	1	1	1	1	1
Maindal [32]	1	1	1	1	1	1	1	1	1	1	1	1
Mok [33]	1	1	1	1	1	1	1	1	1	1	1	1
Sol [34]	1	1	1	1	1	1	1	1	1	1	1	1
Zhang [35]	1	1	1	1	1	1	1	1	1	1	1	1
Daumit [36]	1	1	1	1	1	1	1	1	1	1	1	1

CI, confidence interval; PCE, patient-centered education; RCT, randomized controlled trial.

## Results

### Literature selection

**Figure 1.**
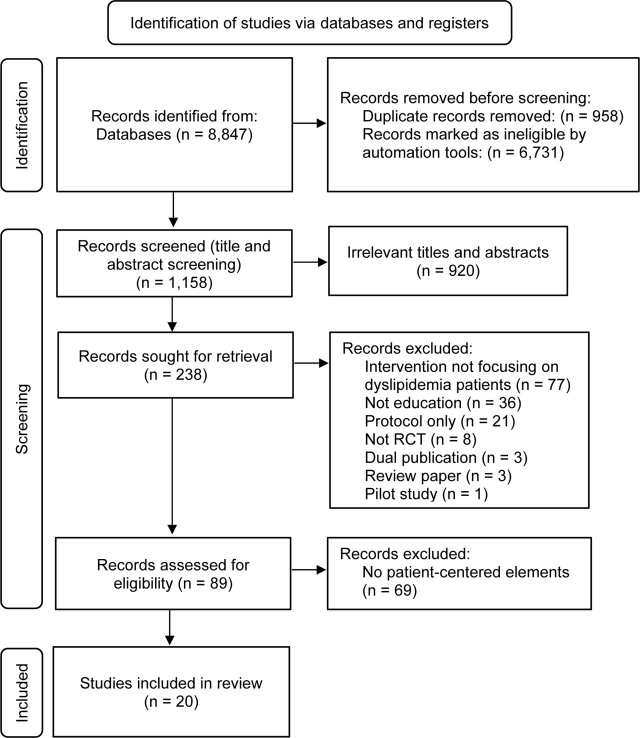
Flow diagram of the study selection process according to PRISMA flowchart. PRISMA, Preferred Reporting Items for Systematic Review and Meta-Analyses; RCT, randomized controlled trial.

**Figure 1** shows the study selection flowchart. Study selection was based on the PRISMA guidelines [14]. We included 20 studies in this systematic review.

### Reviewed studies

**Table 1** summarizes the findings from the 20 publications of the included studies. Fourteen studies were conducted in European countries, 2 studies were conducted in China, and 1 study each in Jordan, Japan, Hong Kong, and the Netherlands. The majority of studies used a 2-arm RCT, and 4 studies used a 3-arm RCT. The number of participants ranged from 62 to 4,105. The intervention’s duration of included studies ranged from 3 months to 48 months.

### Patient-centered approaches

The details of interventions are described in **Table 2**. The interventions involved various health-care professionals, including nurses, pharmacists, physicians, dietitians, kinesiologists, nutritionists, psychologists, and internal medicine specialists. Most of the studies involved nurses in the interventions. Some studies involved single health-care professionals only [3, 19, 20, 30, 31, 33, 34], while others used the collaborative care concept, with ≥2 health-care professionals involved in the interventions. The most used theories in the interventions were the chronic care model, social learning theory, social cognitive theory, transtheoretical theory, health belief model, and MI.

### Outcome measures

All studies reported cardiometabolic outcomes, such as the BP reading, weight, BMI, or HbA1c, except for 4 studies that measured no cardiometabolic parameters [3, 19, 23, 33]. Most of the studies measured cardiovascular risk factors as one of their outcomes, as either primary or secondary outcomes. Cholesterol indices, namely, the TC, TG, LDL, and high-density lipoprotein (HDL) levels, were reported in 18 studies. Some studies also reported psychosocial and cognitive outcomes, such as perceptions of the quality of their chronic illness care [24], patient activation [19, 29], patient-centered care process [29], self-efficacy [34], perceived control and understanding [27], mental health status [6], and quality of life [35]. The majority of studies reported behavioral outcomes, such as consumption of heart-healthy food, physical activity, smoking status, reduction in saturated fat intake, and medication adherence [6, 20,21,22,23, 25, 27, 29, 31,32,33, 35, 36]. Details of the interventions’ impacts are described in **Table 3**.

Based on the studies reported, the findings on cholesterol levels comprised both positive and negative outcomes. Other outcomes measured, such as psychosocial or cognitive outcomes showed promising results in most studies. Improvement in cholesterol level was reported in 11 studies. Significant reductions in all cholesterol-related parameters (TC, LDL, and TG levels), but no significant change in HDL, were found by 2 studies [24, 31]. However, a few other studies [19, 26, 30,31,32,33, 36] reported any 1 of the cholesterol levels (TC/LDL/TG) as improving significantly. Only 1 study reported significant improvement in the HDL level [33]. Most studies reported positive outcomes in the aspect of psychosocial, cognitive, or behavioral outcomes. Significant improvements in other cardiometabolic markers, such as BP, weight, BMI, and HbA1c, were also reported [6, 20, 21, 22, 24, 27, 30, 31, 35].

## Discussion

Overall, based on the reported studies, PCE had successfully improved patients’ cholesterol level, as well as the psychosocial, cognitive, behavioral, and cardiometabolic outcomes. There are a few possible contributing factors that may lead to the effectiveness of the PCE. This discussion is further divided into a few subheadings deliberating on each factor in more detail.

### Underlying theory

Firstly, the desired outcome achieved might be due to the underlying theory used by the researchers. Studies that reported a significant improvement in cholesterol levels (TC/TC/LDL) used MI as one of the key concepts [6, 21, 24, 29, 30, 36]. MI is a communication style that uses specific techniques such as reflective listening, SDM, and eliciting change talk [37]. In addition, the PCE utilizing MI was able to significantly affect the smoking status, which was associated with an improvement in the CVD risk score. Besides smoking status, MI was also beneficial in improving behavioral outcomes, such as physical activity and self-monitoring [21]. Nevertheless, other studies using the MI technique showed no significant improvement in cholesterol levels but achieved significant improvement in psychosocial or cognitive outcomes, such as patient activation [29].

Many confounding factors contribute to the success of the MI intervention, such as health-care professionals’ skills, differences in participants’ characteristics, and time frame of the study. To conduct MI, health-care professionals need to be highly trained. This hypothesis is supported by a study conducted by Allen et al. [24], which suggested that use of MI coupled with appropriate training of health-care professionals would lead to the successful achievement of the desired outcomes. Their study found that using certified nurse practitioners and MI training was associated with significant improvement in psychosocial or cognitive outcomes, cholesterol levels, and measurements of SBP, DBP, and HbA1c [24]. Although MI is a proven and practical method in patient-centered care, as mentioned by Elwyn et al. [16], it is also important for future studies to emphasize the factors that may contribute to the success of the MI, such as the incorporation of health-care professionals’ training.

### Rapid reward system

For behavioral outcomes, many studies reported positive findings in terms of physical activity [6, 21, 25, 27, 31], consumption of healthy foods [6, 20, 25, 31, 33], self-monitoring frequency [23], medication adherence [28, 30, 31], self-care activities [30], and smoking status [36]. Other studies [6, 22, 31, 32, 35] found no significant impact on the smoking status of the participants, except for 1 study by Daumit et al. [36]. This discrepancy may have occurred for a few reasons. A reason for the success of the intervention described by Daumit et al. [36] is the introduction of a point reward system. The investigators successfully educated the patients about smoking cessation, even though encouraging smokers to quit is extremely difficult. In the study, the participants were given points for attendance and behavioral change for smoking cessation. Finally, the points were exchanged with small reward items [36].

This proved that patients might be more motivated by a rapid reward system rather than the promising long-term rewards, such as their health outcomes. This hypothesis is supported by a study by Licthman et al. [38], who described that patients with dyslipidemia are less motivated to improve their cholesterol levels because of the asymptomatic nature of the disease. The motivation of patients with diabetes mellitus is different in that patients can quickly feel the changes in managing their symptoms [38]. Therefore, future studies should incorporate a rapid reward system as an alternative to boost patients’ motivation, especially in promoting behavioral change that demands a lot of effort and sacrifice.

### Dietary education

Consumption of heart-healthy food may lead to significant improvement in HDL levels. However, none of the studies reported significant improvement in HDL levels, except 1 study [33]. It is important to note that improvement in HDL levels could not be achieved solely by taking a lipid-lowering therapy, unlike LDL levels, which decrease with the help of pharmacological treatment. By contrast, HDL level improvement requires a combination of aggressive behavioral interventions, such as a healthy lifestyle, diet, and exercise. The expected significant increment of HDL achieved by Mok et al. [33] was due to the aggressive dietary intervention of the nurses involved in the study. The nurses emphasized appropriate dietary intake and encouraged the patients to eat heart-healthy food. Consequently, there were significant differences in the consumption of saturated fats and healthy food choices. The importance of achieving desirable levels of HDL, TC, LDL, and TG should be emphasized.

### Collaborative care

Collaborative care is a method in which many experts collaborate to improve the quality of health care [39]. Health-care professionals from various disciplines work together as a single unit with centralized communication and coordination to potentially improve patient outcomes [40]. Of the 20 studies included, 12 used collaborative care methods. Goyer et al. [6] involved the highest number of health-care professionals: nutritionists, psychologists, kinesiologists, nurses, and physicians. This collaborative care approach resulted in significant improvement in the psychosocial or cognitive aspect of mental health status. In addition, the behavioral outcomes of calorie intake and physical activities showed significant improvement, together with TG and TC levels.

Ho et al. [28] conducted a study involving primary care clinicians and pharmacists to improve medication adherence among patients with acute coronary syndrome after hospital discharge. The pharmacists provided education and guided the patients to adhere to their medications. The primary care clinicians were then notified about their patients’ medication adherence status using a computerized medical record. The pharmacists’ contact number was also included so that they could be reached for any further questions or clarifications. After 12 months of such intervention, significant improvement in medication adherence was observed [28].

In terms of the pharmacists’ role, medication adherence is one of the crucial components in dyslipidemia management. Medication adherence is an outcome measured in some studies, but the methods of measuring adherence differ between studies. This difference in the method may result in outcome variability. Thus, it is rather challenging to make a generalized conclusion about medication adherence. Some studies used medication refill data from the pharmacy to measure medication adherence [28]. However, the record taken from the pharmacy refill data may be questionable as patients might not take the medication as prescribed, although they did not miss collecting the medications at the pharmacy [39].

Jarab et al. [30] measured medication adherence via self-reports from the patients, and significant improvement was observed. The authors reported that recall bias and social desirability might have occurred during the self-reporting [30]. Despite the disadvantage of self-reporting, Fortin et al. [21] mentioned that this technique is consistent with a patient-centered approach. Byrne et al. [27] measured medication adherence using a biochemical urine test and found no significant differences between groups. A biochemical urine test identifies the level of statin in the urine and is only applicable to test for atorvastatin [27].

Collectively, various methods of adherence measurement may affect the findings. To date, measuring medication adherence by using a biochemical urine test seems to be one of the alternatives. However, information regarding the cost and time constraints for implementing this method is scarce [27]. Various methods of measurement, such as self-reporting, records taken from pharmacy refill data, or biochemical urine tests, have their advantages and disadvantages. In highlighting the pharmacist’s role, future studies in measuring medication adherence should be conducted based on resource availability, drugs involved, and patients’ preferences.

The success of collaborative care in the interventions described earlier by 2 studies [6, 28] was associated with focused education content based on the health-care professionals’ expertise. The collaborative care or multidisciplinary approach should be extended to patients with CVD to optimize their care and risk reduction [6]. Future studies may consider the element of collaborative care to optimize the roles of different health-care professionals who have similar aims in improving patients’ overall clinical outcomes.

### Duration of intervention and systematic follow-ups

Duration of intervention can contribute to the efficacy of PCE. PCE has been used to reduce CVD risk among patients [36]. Significant improvement in smoking status was observed after 18 months. The effectiveness of the intervention, which leads to behavioral change, is possibly due to its longer duration. An individualized counseling session was conducted weekly or every 2 weeks. Systematic follow-ups played an important role in determining the effectiveness of the intervention. The time frame of the reported studies varies between 2 months and 4 years. In one of the studies reported by Maindal et al. [32], there was no significant difference in the CVD risk levels after 3 years of interventions. Although outcomes were measured after 3 years, there were no systematic follow-ups implemented before measuring the outcomes. Therefore, the authors [32] suggested providing gradual sessions at 1 month, 2 months, and 3 months after the core interventions.

Mok et al. [33] emphasized that systematic follow-ups are important. The authors highlighted that follow-ups conducted via telephone calls were a key component that led to significant positive dietary changes. The telephone calls were made fortnightly for the 2-month duration [33]. This assertion is supported by Jarab et al. [30], who found significant differences during the 6 months of their study duration. However, they were unsure of the impact of the intervention beyond that time [30]. Future studies should be conducted to explore the minimum duration of follow-up needed to sustain the desired outcomes. Systematic follow-ups, either face-to-face or by telephone calls, are crucial to ensure the effectiveness of the intervention.

### Social support

The role of family members throughout the intervention period has been highlighted [31]. Recruitment of family members can encourage social support for the patients [41]. If there is no support available through a family, finding supporting individuals or groups is important for the patients to initiate behavioral change [41]. Dunbar et al. [42] asserted that social support is associated with better CVD outcomes. They indicated that family support is linked with the adoption and maintenance of healthy behaviors.

Kitko et al. [43] emphasized the role of caregivers in individuals with heart failure. They highlighted that many individuals with heart failure depend on support from their partners, families, friends, or neighbors to help them manage chronic diseases. Strom and Egede [44] also asserted that social support also significantly improves patients’ motivation. Therefore, the active involvement of family members or other individuals to provide social support to the patients throughout the intervention is strongly encouraged.

### Usage of e-health

E-health has been discussed in the literature for more than a decade. E-health is an emerging field that refers to health services and information that are conveyed or enhanced through the Internet or related technologies [45]. Snyder et al. [46] mentioned that information technology is able to promote patient-centered care by providing a mechanism for the patient to share their information with health-care professionals. Wong et al. [47] reported that online counseling might serve as an alternative to reach those who remain untreated.

Moessner and Bauer [48] found that an Internet-based service reached a substantial proportion of individuals with eating disorders. More than half of the participants (57.3%) said that the Internet was their first choice for seeking professional assistance. The participants indicated that they would not have shown behavioral changes had it not been for the online services [48]. Many articles have explored online counseling for mental health patients, but limited research has been done on managing CVD patients.

Of the 20 articles included in this review, only 1 study described an e-health intervention [3]. Despite the promising outcomes of this intervention, Eaton et al. [3], who conducted a study on utilizing e-health or electronic devices to improve cholesterol management, did not find any significant improvement in any of the outcomes. The authors considered that the e-health tools might be improved in the future by making them more user-friendly. Before 2011, when the study was conducted, the patients might not have been familiar with technology and might have found it difficult to use, resulting in minimal benefits. Today, people are more familiar with technology, especially after the coronavirus disease-2019 (COVID-19) pandemic. O’Leary [49] found that there was a rapid demand for efforts to use technology to cope with the damage caused by COVID-19. This opportunity should be utilized by the health-care system, whereby online methods should be implemented as much as possible when appropriate. When more people are able to adapt to technology, the delivery of technology-assisted online counseling continues to grow [47]. Therefore, looking at the opportunity of available technology and readiness of patients, education using online platforms may produce a promising result. Online counseling may replace telephonic or face-to-face counseling for systematic follow-ups for patients with CVD and help particularly in managing their dyslipidemia. Online counseling also may enhance the patient–doctor relationship, which is a dimension in the patient-centeredness concept highlighted by Stewart et al. [12]. However, the performance of the utilized software and digitalized platforms should be carefully examined to ensure that they can achieve the objective of patient-centered care.

## Conclusion

The use of PCE is beneficial and superior to the usual form of care for managing dyslipidemia. Most studies reported successful findings in terms of either clinical outcomes (cholesterol level) or psychosocial, cognitive, or behavioral outcomes. Underlying theory, the rapid reward systems, dietary education, collaborative care, duration of intervention with systematic follow-ups, social support, and usage of e-health platforms have important roles in achieving desired outcomes.

To our knowledge, this review is the first to report the effectiveness of PCE for patients with dyslipidemia. The studies that were included in the review were methodologically sound and confirmed with CASP. Our review is comprehensive as it reports not only clinical outcomes in terms of cholesterol levels but also includes psychosocial, cognitive, behavioral, and other cardiometabolic parameters. This enabled the reviewers to analyze how the interventions may affect 1 or all of the desired outcomes being measured. This review also extracted patient-centered elements, other than education, which would be helpful in future studies.

However, this review also has a few limitations. First, we integrated research with a variety of interventions, measures, and follow-up times, which made it challenging to synthesize clear conclusions. Study designs and outcomes may have produced a more consistent finding if we had focused on smaller groups. However, this approach would have limited the number of studies we may have included. Some of the research works included in this review were conducted with specific target groups that may not be generalizable to all populations. Therefore, our findings should be interpreted with caution.

Second, there are many confounding factors that may affect the success of the interventions, such as the health-care professionals’ training, patient demographics and characteristics, their financial status, and social support that they received. All these will eventually affect the outcomes measured after the interventions. Hence, the findings may not be generalized, and the results should be extrapolated with caution. Third, there is a possibility of medication changes made by the primary physicians in each study, but not being reported. This could potentially affect the final results. This limitation serves as a guide for future studies to include all interventions made throughout the study to enable more conclusive findings. Finally, the outcomes reported were not consistent in all included studies. The majority of the studies reported the LDL levels, whereas other studies reported other cholesterol indices. Complete cholesterol indices comprising TC, TG, LDL, and HDL levels would help to provide clearer conclusions.

Despite these limitations, the present review describes the effects of PCE compared with usual care or didactic/traditional forms of education. It is also hoped that the present review will provide guidance for patient-centered intervention for health-care professionals in managing patients with dyslipidemia.
